# The comparison of anxiety and depression rate between medical staff of infertility centers and obstetrics and gynecology centers of Yazd, Shiraz, Isfahan and Kerman hospitals

**Published:** 2014-03

**Authors:** Zahra Pourmovahed, Seyed Mojtaba Yassini Ardekani, Mohammad Ali Khalili, Iman Halvaei, Ali Nabi, Mojdeh Ghasemi, Farzaneh Fesahat

**Affiliations:** 1 *Department of Nursing Education, Shahid Sadoughi University of Medical Sciences, Yazd, Iran.*; 2 *Department of Psychiatry, Shahid Sadoughi University of Medical Sciences, Yazd, Iran.*; 3 *Research and Clinical Center for Infertility, Shahid Sadoughi University of Medical Sciences, Yazd, Iran.*; 4 *Fertility and Infertility Research Center, Isfahan University of Medical Sciences, Isfahan, Iran.*

**Keywords:** *Anxiety*, *Depression*, *Infertility departments*, *Obstetrics and gynecology departments*

## Abstract

**Background:** Regarding the close and continuous interaction of infertility staff with hopeless infertile couples and in the contrary the atmosphere of happiness especially in obstetric wards make a sense that considering anxiety and depression it would be a difference between these two wards.

**Objective:** The objective of this study is the comparison of the rate of depression and anxiety between the two wards of infertility and obstetrics and gynecology.

**Materials and Methods: **This study is a descriptive-correlation study based on cross-sectional method. 199 individuals who were the staff of infertility and obstetrics and gynecology wards in four provinces enrolled in this study through stratified sampling. Data collection was done by demographic questionnaire, Spiel Berger and Beck depression inventory tests. Data were analyzed by SPSS software using ANOVA test.

**Results: **The result showed the rate of anxiety in obstetrics and gynecology staff of Isfahan center (54.69±13.58) and depression rate had increased level in infertility staff of Shiraz center (14.94±10.87). Overall, there was significant correlation between anxiety, depression and work place (p=0.047, 0.008 respectively). According to ANOVA test, the mean value of anxiety level was higher in the staff of four obstetrics and gynecology centers and one infertility center

**Conclusion:** As long as we know that infertile couples have little chance for success rate and obstetrics and gynecology wards patients have little risk of failure in treatment, it could be mentioned that the anxiety and depression in the staff are not correlated with the client illness.

## Introduction

Mental health is an important issue in the personal, social and occupational function of everyone and not caring this issue would cause decrement or loss of efficacy, manpower staffing and negative consequences on professional health services (1). One of the most important parts of community health development is the health and management discipline which is in close relationship with the human health and working in these disciplines has been known as high stress job (2, 3). Mental problems such as anxiety and depression may cause decrement of motivation, interest and impaired function and comorbidity of anxiety and depression make the problem worse (4, 5). Kaplan showed that general health questionnaire (GHQ) core in 47% of physicians, manager and hospital consultants of North Lincolnshire was more than normal. This report indicate that an increase in anxiety and depression rate among the medical staff (6). 

Studies on the hospitals' staffs indicating that some factor like the high load of the work, lack of appropriate support, frequent contact with patients, lack of cooperation and misunderstanding between patients, staff and their families make the staff susceptible to psychiatric morbidity (3, 7, 8). Considering these issues, any attempt for the maintaining or promotion of the mental health of the staffs would make them more efficient and productive. Beside the infertile couples usually suffer a psychiatric morbidity and as long as supporting the clients are part of the staff responsibility, the mental health of the staff needs to be kept in mind. 

Regarding to the close interaction of the infertility center staff with hopeless infertile couples we propose that staff of infertility centers have to be more depressed or anxious than obstetrics and gynecology ward staff 

## Materials and methods

This study is a correlation study based on cross sectional method for evaluation and comparison of the anxiety and depression rate in the staff of hospitals and infertility centers in 1391. To do stratified sampling, first of all the staff population was estimated in infertility and obstetrics and gynecology centers of four centers and samples were statistically gathered based on the population of these centers by considering the p-value of 5%, statistical power of 80% and previous studies, 199 individuals were calculated as sample size. Demographic data obtained through questionnaire and structured interview by a trained and qualified person. 

We used content validity index to determine the validity of questionnaire. So the questionnaires were evaluated according to the research objectives by a few experts. In order to assess reliability, 20 questionnaires were distributed and Cronbach's alpha coefficient was calculated. Anxiety and depression rate was determined through Spiel Berger scale (trait) and Beck depression inventory test respectively. 

Inclusion criteria were working in obstetrics or gynecology ward for at least one year and anyone who had a visit for psychiatrist, excluded from the study. All participants signed the informed consent. Spiel Berger scale consists of 20 questions which are scored from 1-4 and Beck inventory test consists of 21 items score ranging from 0-3. This study was approved by the ethical committee of infertility center of Yazd and financially supported by Shahid Sadoughi University of Medical Sciences. The researchers assured participants that all information is confidential.

**Statistical analysis**

The data were presented as mean±SD. Encoded data was analyzed by SPSS software version 16 (Chicago, USA) using ANOVA test. P-value of 0.05 or less was considered as statistically significant.

## Results

Out of 199 participants, 88.9% were female and 11.1% were male. 73.4% of them were married, 26.4% single and 2% were divorced or widowed. The mean age was 34.62±8.02 with the average amount of work experience of 9.55±7.19 years. The most of subjects had completed bachelor science degree (58.8%). The mean value of anxiety and depression score was 42.23±10.64 and 10.65±8.34 respectively. The results revealed no significant relationship between age, sex, marital status and duration of work experience with anxiety or depression. According to table I, the participants with associate’s degree showed the highest mean value of anxiety (46±12.12) and depression (14.36±7.48). There was just a significant correlation between depression and education level (p=0.03).

The result showed that anxiety has increased level in obstetrics and gynecology staff of Isfahan center (54.69±13.58) and depression in fertility staff of Shiraz center (14.94±10.87). Overall, correlation between anxiety, depression and place of work was significant statistically (p= 0.047, 0.008 respectively) (Table II). Also the data showed that mean anxity score was significantly higher in obstetrics and gynecology ward of Isfahan, Shiraz, Shahid Sadoughi and Yazd Madar centers (p=0.03, 0.006, 0.008, 0.012 respectively) and also the infertility center of Yazd (p=0.00). 

Table III indicates that the highest mean score for anxiety was 47.50±19.15, and 46±11.13 in secretary working in OB&GYN and infertility centers and anesthesia technician and depression rate was higher in anesthesia technician, respectively (16.66±5.13) in comparison with the rest of staff, although there was just a significant correlation between anxiety and job category by ANOVA test (p=0.031).

**Table I:** Anxiety and depression scores in staff working at infertility centers and obstetrics and gynecology wards based on the education level

**Education level**	**N (%)**	**Anxiety score** *	**Depression score** *
Diploma	23 (11.6)	44.69 ± 10.34	13.3 ± 11.8
Associate degree	19 (9.5)	46 ± 12.12	14.36 ± 7.48
Bachelor science	117 (58.8)	33.42 ± 10.4	10.47 ± 8.13
Master science	15 (7.5)	37.8 ± 11.3	7.33 ± 6.06
Philosophy doctor	25 (12.6)	39.28 ± 9.56	8.24 ± 6.74
Total	199 (100)	42.23 ± 10.64	10.65 ± 8.34
		p=0.08	p=0.03

* Data are presented as Mean±SD. ANOVA test

**Table II:** Anxiety and depression scores in medical staff based on provincial obstetrics and gynecology wards and infertility wards

**Infertility centers and obstetric and gynecology wards**	**n(%)**	**Anxiety score** *	**Depression score** *
Shiraz infertility center	19 (9.5)	41.68 ± 10.74	14.94 ± 10.78
Dena infertility center	7 (3.5)	39.71 ± 7.71	9.85 ± 5.14
Ghadir infertility center	6 (3)	40.33 ± 9.89	7.66 ± 5.16
Shiraz obstetric and gynecology ward	18 (9)	40 ± 8.61	6.05 ± 6.5
Yazd infertility center	40 (20.1)	39.57 ± 10.35	9.32 ± 7.71
Shahid Sadoughi obstetric and gynecology ward, Yazd	30 (15.1)	41.5 ± 11.32	11.76 ± 8.38
Madar hospital obstetric and gynecology ward, Yazd	27 (13.6)	41.59 ± 9.22	12.29 ± 8.34
Isfahan infertility center	22 (11.1)	44.04 ± 9.49	13.18 ± 10.28
Isfahan obstetric and gynecology ward	13 (6.5)	54.69 ± 13.58	8.46 ± 6.87
Kerman infertility center	9 (4.5)	44.77 ± 10.84	10.66 ± 6.14
Kerman obstetric and gynecology ward	8 (4)	42.25 ± 8.01	7.25 ± 4.65
Total	199 (100)	42.23 ± 10.64	10.65 ± 8.34
		p=0.008	p=0.047

* Data are presented as Mean±SD. ANOVA test

**Table III:** Anxiety and depression scores in staff working at infertility centers and obstetrics and gynecology wards

**Occupational Level**	**Anxiety score**		**Depression score**	
	**n(%)**	**Mean ± SD**	**n(%)**	**Mean ±SD**
Gynecologist	10 (5.2)	38.9 ± 8.15	10 (5.2)	7.4 ± 5.44
Obstetrician	51 (26.6)	44.52 ± 10.26	51 (26.6)	11.64 ± 8.21
Nurse	64 (33.3)	43.18 ± 10.02	64 (33.3)	10.48 ± 8
Operating RoomTechnician	5 (2.6)	36.2 ± 9.54	5 (2.6)	10.2 ± 7.88
AnesthesiaTechnician	3 (1.6)	46 ± 11.13	3 (1.6)	16.66 ± 7.13
Laboratory Technician	24 (12.5)	37.0 ± 10.07	24 (12.5)	8.91 ± 7.55
Secretary	4 (2.1)	47.5 ± 19.15	4 (2.1)	12.5 ± 10.4
Assistant	21 (10.9)	44.04 ± 10.97	21 (10.9)	13.61 ± 11.64
Physician	10 (5.2)	43.6 ± 10.61	10 (5.2)	10 ± 7.27
Total	192 (100)	42.28 ± 10.62	192 (100)	10.69 ± 8.35
		p=0.031		p=0.401

ANOVA test.

## Discussion

The results showed that anxiety was moderate (42.23±10.64) in obstetrics and gynecology staff and infertility centers. Moreover, depression was not common. According to our data, anxiety was higher in obstetrics and gynecology staff of Isfahan center (54.69±13.58) and depression in infertility staff of Shiraz center (14.94±10.87) comparing to the rest of staff. Anxiety was the most common disorder (11.4%) that was reported by Sadeghi *et al* (1). Anxiety may be caused by different factors including stressful work conditions, high workload, unpredictable events and also individual factors which are reported in previous studies (2, 3, 7, 8). 

Khaghanizadeh *et al* also mentioned that anxiety is the most common mental disorder in medical staff (9). Sahebi and Ayatollahi reported that 45.6 % of medical staff at Shiraz hospitals suffering mental problem (10). Kaplan showed that GHQ score in 47% of physicians, manager and hospital consultants of North Lincolnshire was more than normal range that is indicative of increased anxiety and depression rate among the medical staff (6). 

In this report, there was no statistically significant relationship between age, sex, marital status and duration of work experience with anxiety or depression which is in consistent with Hashemi Nazari *et al* report (13). Otherwise, our results revealed no similarity with Saberin *et al*, Bigdeli and Karimzadeh research (12). Finding that should be considered, is the high number of female staff in these centers which was 88.9% in the present study. Women have other responsibilities in addition to their jobs such as house holding and child training which make more stress and more chance of developing mental problems. In the present study, mean score of anxiety and depression was 42.93±10.37, 10.96±8.13 respectively in female subjects although there was no significant correlation between sex and anxiety or depression. The participants with associate’s degree showed the highest anxiety (46±12.12) and depression (14.36±7.48). There was just a significant correlation between depression and education level (p=0.030). In our study, increasing education level was associated to decreased anxiety and depression which is in consistent with Lambert *et al* and Arasteh’s study(14, 15). 

Higher level of education and income could cause less stress and more security and life expectancy and social promotion of anyone could be affected by education level and job position. The anxiety in different groups including secretaries, anesthesia technicians, midwives, health workers and nurses was higher comparing to other medical staff which is indicative of significant correlation between anxiety and job position (p=0.031). But there was no significant correlation between job position and depression although depression was found more prevalent in anesthesia technicians (16.66±5.13). High anxiety in secretaries may be explained by their close interaction with clients, who are anxious, hopeless and have not enough patience. Whilst, being supportive is part of the staff responsibility and as long as other studies indicate high anxiety level in medical staff, looking for a problem solution is indicated (16). 

Regarding to high anxiety level in staff that working at Isfahan obstetrics and gynecology centers, it could be said that they have less inhibition in expressing of their feeling due to their culture. Regarding the feelings of the staff at shiraz center, it could be due to other factors like work place, interactions, administration and management rules and nowadays, it is recommended to consider all the factors related to in-and outside the work environment in assessment of mental disorders (17). It seems that organizational factors play an important role like workload, work time and job security (2, 18). Regarding to the effect of anxiety in decreasing concentration and developing forgetfulness, the chance of unwanted events and error in doing job duties would be increased memory and it is recommended that managers to become aware of the importance of these kind of psychiatric morbidity and their negative consequences.

Finally by admitting the high level of anxiety in these four provincial medical centers we have to looking for some therapeutic strategies to manage the present anxiety and some policies for prevention of anxiety development such as job security, having much income, setting up the consultation services and even tourism facilities. We have to mention that the main limitation in this study was the lack of cooperation in some staff and specially physicians specialist.

## Conclusion

Since there was no significant difference in the level of staff anxiety in obstetrics and gynecology staff and infertility wards of four provincial centers except Isfahan, it could be proposed that high level of anxiety might be due to other factors such as administration, management policies and interpersonal interaction in different work place. Depression showed increased level in the staff of Shiraz infertility center and it could be justified like anxiety causes.
